# Prognostic value of ErbB2/HER2 in human meningiomas

**DOI:** 10.1371/journal.pone.0205846

**Published:** 2018-10-18

**Authors:** Magnus B. Arnli, Theo L. Winther, Stian Lydersen, Sverre H. Torp

**Affiliations:** 1 Department of Clinical and Molecular Medicine, Faculty of Medicine and Health Sciences, Norwegian University of Science and Technology (NTNU), Trondheim, Norway; 2 Regional Centre for Child and Youth Mental Health and Child Welfare, Department of Mental Health, Faculty of Medicine and Health Sciences, Norwegian University of Science and Technology (NTNU), Trondheim, Norway; 3 Department of Pathology, St. Olavs Hospital, Trondheim, Norway; University of South Alabama Mitchell Cancer Institute, UNITED STATES

## Abstract

**Introduction:**

Among clinical challenges regarding human meningiomas is their propensity to recur even in cases with benign histology. Reliable biomarkers that can identify these cases are therefore highly desired. ErbB2/HER2 status is important in the medical management of patients with various human malignancies, whereas its clinical relevance in human meningiomas is ambiguous. For this reason, we wanted to investigate the expression of intra- and extracellular domains of ErbB2/HER2 as well as the level of activated receptor in these tumors. Further, we wanted to elucidate any clinicopathological associations to antibody expression and if gene amplification was present.

**Methods:**

In total, 186 human meningiomas of all malignancy grades were included in the study, 163 of these were in tissue microarrays (TMA). Antibody expression was assessed by means of immunohistochemistry (IHC) and gene amplification by fluorescence in situ hybridization (FISH).

**Results:**

All cases were immunoreactive with antibodies targeting the intracellular domain, whereas about 48% and 11% were positive with antibodies against the extracellular domain and against the activated receptor, respectively. Normal meninges were not immunoreactive. There were no relations to malignancy grade, and only the activated receptor was significantly correlated with increased risk for recurrence or death (time to recurrence: HR 1.568, CI (1.153 to 2.132), p = 0.004). No gene amplification was found.

**Conclusion:**

ErbB2/HER2 is generally upregulated in human meningiomas, but in an activated state only in a few cases. Only the activated receptor is associated with poorer prognosis, a link that needs further investigations.

## Introduction

Meningiomas are the most common benign intracranial tumors in humans. They originate in the arachnoid layer of the meninges and may accordingly afflict the central nervous system in a number of locations, including the spine [1]. Meningiomas more often afflict women than men and are rare but often aggressive in children [1, 2]. Most meningiomas in adult patients are benign, yet a substantial number of such tumors recur [3]. Thus, identifying recurrence-prone cases is of major clinical importance, and search for relevant and reliable biomarkers is required.

ErbB2/HER2/neu (from now called HER2) is a tyrosine kinase receptor belonging to the EGFR family that lacks specific ligands [4, 5]. It is the dominant ErbB dimerization partner most likely due to its constitutively open conformation, and when present, there is a strong tendency of heterodimer formation [5, 6]. It may also activate by forming homodimers without ligand stimulation in HER2 overexpressing tumors [5]. The ErbB-receptors contain an extracellular domain that exposes a “dimerization arm” upon ligand stimulation, allowing for hetero- or homodimerization [4, 6]. Further, the ErbBs contain a transmembrane domain and an intracellular kinase-containing domain that participates in phosphorylation and initiation of intracellular signaling, leading to apoptosis, growth, migration, adhesion, and differentiation [4, 5]. Gene amplification and overexpression of HER2 has been reported in for instance gastric- and endometrial cancers and urothelial bladder carcinoma [7], yet its clinical significance is perhaps best documented in breast cancer where these events have therapeutic implications [8].

Expression of HER2 has previously been investigated in human meningiomas with ambiguous results [9]. Low [10, 11], moderate [12, 13], and large [14–19] proportions of immunoreactive tumors have been reported. Regarding *HER2* gene amplification, this has been detected in a minority of cases [13], while others have failed to identify this feature [14, 15, 17]. Few studies have investigated the clinicopathological aspects of HER2 in meningiomas. Some have found an inverse association between increased HER2 expression and tumor grade [17], and others no association [19]. However, the prognostic value of this receptor protein remains unclear [10, 13]. Partly, the discordance in the existing literature could be due to the use of antibodies targeting either extracellular (ECD) or intracellular (ICD) domains of the receptor. Considering that the HER2 ECD may be shedded, resulting in a truncated receptor (p95HER2) [6], this is an important issue that needs to be addressed.

The aim of this study was to evaluate the expression and amplification of *HER2* in a large series of consecutively operated human meningiomas in adult patients and relate these findings to prognosis. The existing literature demonstrates heterogeneous results on this issue and is complicated by use of various antibodies targeting different domains of the receptor. The current study seeks to elaborate on these findings by presenting results for several antibodies on nearly two hundred meningioma specimens. In this endeavor, expression levels of the extra- and intracellular domains of HER2 and of the activated receptor were investigated. In sum, variable expression patterns were observed for the different antibodies and the amplification status was successfully addressed. Additionally, the activated HER2 receptor may have prognostic significance.

## Materials and methods

### Specimen selection and TMA construction

Collection of cases, histopathological examination, and tissue microarray (TMA) construction as well as the clinicopathological data have been described in detail in previous studies [20–22]. Shortly, adult patients over 18 years of age consecutively operated for meningioma at St. Olavs Hospital, Trondheim, Norway over a ten-year period (1991–2000) were evaluated for inclusion. The tumors were fixed in formalin and embedded in paraffin after operation and were recently reviewed and graded according to the WHO 2016 criteria [23]. In total 186 meningioma specimens were included in this study, 163 of which were prepared as TMAs. Representative tumor tissue for TMA inclusion as seen on hematoxylin/eosin (HE) sections was defined as “meningioma tissue lacking necrosis and with minimal connective- and vascular tissue, hemorrhages, and calcifications” [21]. The TMAs consisted of tumors with three extracted cores for each case and were created with an Alphelys Arrayer Minicore 3, AH diagnostics, with TMA Designer2 software. TMA sections were scanned and analyzed on electronic images (Genetix, Ariol SL-50 3.3). The remaining 23 cases were regarded unfit for TMAs due to insufficient tissue mass, and therefore whole-tissue sections were prepared and analyzed using a Nikon Eclipse 50i microscope.

### Immunohistochemistry

Four-micrometer thick paraffin sections were dried over night at 37°C and stored in a freezing unit. Sections were heated at 60°C for one hour, deparaffinized and rehydrated, and later pre-treated for antigen retrieval with PT Link (Dako). Endogenous peroxidase activity was quenched over 10 minutes with diluted hydrogen peroxide. Primary antibodies included clone CB11 (intracellular domain, monoclonal mouse, pH 9, 1:40 dilution, c-erbB-2 Oncoprotein, cat# NCL-L-CB11, Novocastra, Leica Biosystems), clone 3B5 (intracellular domain, monoclonal mouse, pH 6, 1:10 dilution, Anti-ErbB 2 antibody, cat# ab16901, Abcam), clone SP3 (extracellular domain, rabbit monoclonal, pH9, 1:10 dilution, HER2/ErbB 2 Antibody, cat# MA5-16348, Thermo Scientific), and clone 6B12 (phosphorylated intracellular domain, rabbit monoclonal, pH 9, 1:10 dilution, Phospho-HER2/ErbB2, Tyr1221/1222, cat# 2243, Cell Signaling Technology) and these were incubated for 60 minutes using a Dako AutoStainer Plus. Secondary antibodies with horseradish peroxidase were incubated for 30 minutes (Dako EnVision). Diaminobenzidin (DAB) was used as chromogen and hematoxylin as counterstain. A staining index (SI) was calculated as a product of intensity and fraction of positive tumor cells [24]. Intensity was recorded as 0 (no reaction), 1 (weak), 2 (moderate) or 3 (strong), and the fraction of positive tumor cells as 0 (no positivity), 1 (< 10% positive cells), 2 (10–50% positive cells), or 3 (> 50% positive cells). Non-neoplastic meninges found in a subset of meningioma specimens were also examined for antibody expression. Paget’s disease of the nipple, skin or adenocarcinoma (lung) was used as positive controls for each staining run (S1 Fig). Two of the authors (MBA and TLW) separately analyzed each case on either electronic images or in the microscope, and discrepancies were resolved by an experienced neuropathologist (SHT).

### Fluorescence in-situ hybridization (FISH)

Amplification of *HER2* was assessed using the Dako IQFISH PharmDx assay protocol for breast cancer. In total, 186 tumors were investigated for amplification using a Nikon Eclipse 90i with Cytovision software. HE-sections were examined simultaneously through a conventional microscope to confirm evaluation of tumor tissue. DAPI was used to stain tumor cell nuclei. The centromere at chromosome 17 (CEN-17) was used as reference and stained with fluorescein-tagged CEN17 PNA-probes, while *HER2* was stained with Texas Red. The evaluation procedure followed the Dako interpretation guide. Specifically, different regions of the tumors were evaluated to compensate for potential tissue heterogeneity, and only isolated, non-overlapping cells were counted. In TMA tumors, all three cores were inspected. After counting 20 different cells, a *HER2*:CEN-17 ratio was calculated from the sums of each signal. *HER2*:CEN-17 > 2 was defined as amplification.

### Statistical analysis

HER2 SI was compared across malignancy grades using Mann-Whitney U tests. The interrater agreement was assessed with a weighted kappa statistic (StatXact 11). Tumor localization and subtypes were tested for any relation to HER2 SI using the Kruskal-Wallis test. Significant omnibus tests were planned to be succeeded by pairwise comparisons with Dunn’s test and Hommel adjustment, but none of the omnibus tests were significant. Survival analyses based on continuous SI values were performed with the Cox proportional hazards model, and significant results were adjusted in multivariable analyses including malignancy grade (grade I vs grades II and III), Simpson resection grade (I and II vs III and IV), WHO performance status (0 and 1 vs 2, 3, 4, and 5), and age at operation (continuous values) as covariates. The proportional hazard assumption was checked by visual inspection of log-log plots. Time to recurrence (TTR, defined as time to recurrence or disease related death) [25] and overall survival (OS) were chosen as endpoints. Patients were followed for a maximum of eighteen years (OS) or fifteen years (TTR). Two-sided p-values <0.05 were regarded as significant. SPSS version 24.0 (SPSS Inc., Chicago, IL) was used in statistical analyses.

### Ethical approval

The study has been approved and a waiver of consent was given by the Regional Committees for Medical and Health Research Ethics (REK) (project number 4.2006.947).

## Results

### Patient data

The patients in this study (n = 186) comprised 138 females and 48 males, with a F:M of 2.9:1. The median age at the time of operation was 59 years. Tumor localizations included the convexity (86), basis cranii (47), posterior fossa and tentorium (28), falx (24), and intraventricular (1). Most tumors underwent gross total resection: Simpson grade I (44), grade II (80), grade III (30), and grade IV (32). According to the WHO 2016 grading criteria, there were 129 benign tumors (grade I), 56 atypical (grade II), and one anaplastic (grade III). Patient data across WHO grades are presented in Table 1.

**Table 1:** Patient data.

Patient data	All Grades	Grade I	Grade II	Grade III
Cases	n = 186	n = 129	n = 56	n = 1
Age at operation: mean (SD) and median (min-max)	59.28 (13.28) 59 (25–86)	58.69 (13.24) 58 (27–84)	60.46 (13.47) 62 (25–86)	69
Sex male/female	48/138	29/100	18/38	1/0
Simpson grade I/II/III/IV	44/80/30/32	33/53/18/25	11/27/11/7	0/0/1/0
WHO Performance Status 0/1/2/3/4	25/130/27/3/1	18/89/20/2/0	6/41/7/1/1	1/0/0/0/0
Localization falx/convexity/basal/posterior fossa and tentorial/intraventricular	24/86/47/28/1	13/51/42/23/0	11/34/5/5/1	0/1/0/0/0

### Immunohistochemical and hybridization analyses

**Fig 1:**
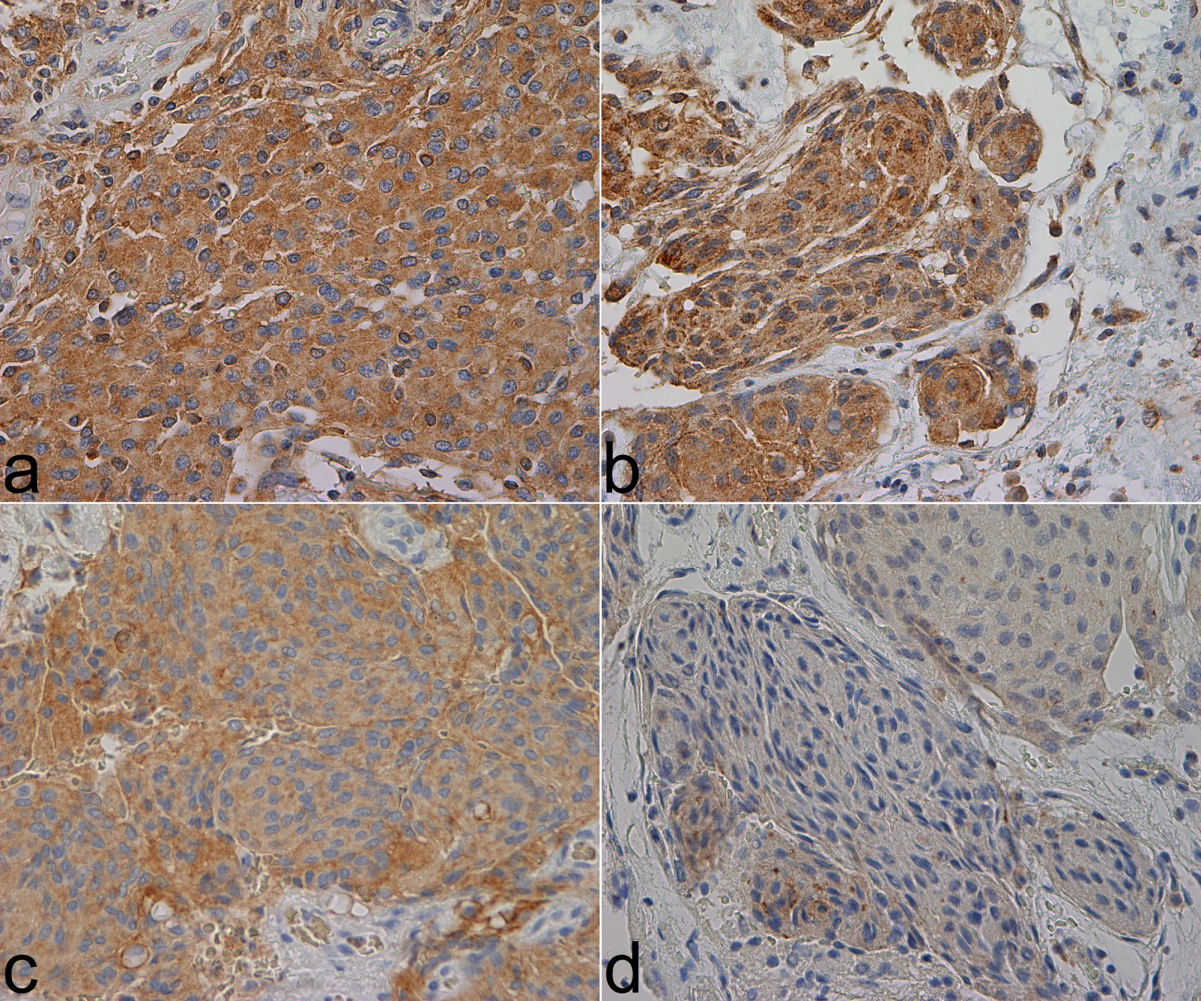
HER2 immunostainings. The pictures were taken with a Nikon eclipse 80i microscope, Lumenera’s Infinity2 camera, and Infinity Analyze software. Magnification: 400x. Microsoft Paint was used to create the final composite image. a. CB11, SI 9 b. 3B5, SI 9 c. SP3, SI 6 d. 6B12, SI 3.

Immunohistochemical data are presented in Table 2. One case was lost due to an inferior amount of tissue during the analysis of CB11 and 6B12. The mean SI values were high for both CB11 and 3B5 (7.86 and 8.34, resp.), but were mostly low or negative for SP3 and 6B12 (1.34 and 0.22, resp.). 48.4% and 10.8% of the cases were positive for SP3 and 6B12, respectively. CB11 and 3B5 were positive in all cases, and at times exhibited a granular staining pattern. All antibodies exhibited predominantly cytoplasmic immunoreactivity (Fig 1). Membranous reactivity occurred as well but was not as pronounced and more difficult to assess. Endothelial cells and non-neoplastic meningeal tissues were immunonegative. Tumor localization and histological subtype were not associated with any antibody expression (S1 Table). None of the antibodies displayed significant relation to tumor grade (Table 3).

**Table 2:** Immunohistochemical results.

**CB11 (ICD)**	**Measure**	**All grades** **n = 185**	**Grade I** **n = 129**	**Grade II** **n = 55**	**Grade III** **n = 1**
	Percent positive	100	100	100	100
	Median SI (min-max)	9 (3–9)	9 (3–9)	9 (3–9)	9
	Mean SI	7.86	7.91	7.75	9.00
**3B5 (ICD)**	**Measure**	**All grades** **n = 186**	**Grade I** **n = 129**	**Grade II** **n = 56**	**Grade III** **n = 1**
	Percent positive	100	100	100	100
	Median SI (min-max)	9 (3–9)	9 (3–9)	9 (3–9)	9
	Mean SI	8.34	8.22	8.63	9.00
**SP3 (ECD)**	**Measure**	**All grades** **n = 186**	**Grade I** **n = 129**	**Grade II** **n = 56**	**Grade III** **n = 1**
	Percent positive	48.4	51.9	41.1	0
	Median SI (min-max)	0 (0–6)	1 (0–6)	0 (0–6)	0
	Mean SI	1.34	1.47	1.05	0
**6B12 (pHER2)**	**Measure**	**All grades** **n = 185**	**Grade I** **n = 129**	**Grade II** **n = 55**	**Grade III** **n = 1**
	Percent positive	10.8	11.6	9.1	0
	Median SI (min-max)	0 (0–3)	0 (0–3)	0 (0–3)	0
	Mean SI	0.22	0.23	0.20	0

**Table 3:** Differences in staining index by malignancy grade (p-values, 2-tailed exact values from Mann-Whitney U tests).

	CB11	3B5	SP3	6B12
**Grade II vs grade I**	0.659	0.085	0.111	0.689

n = 185.

**Fig 2:**
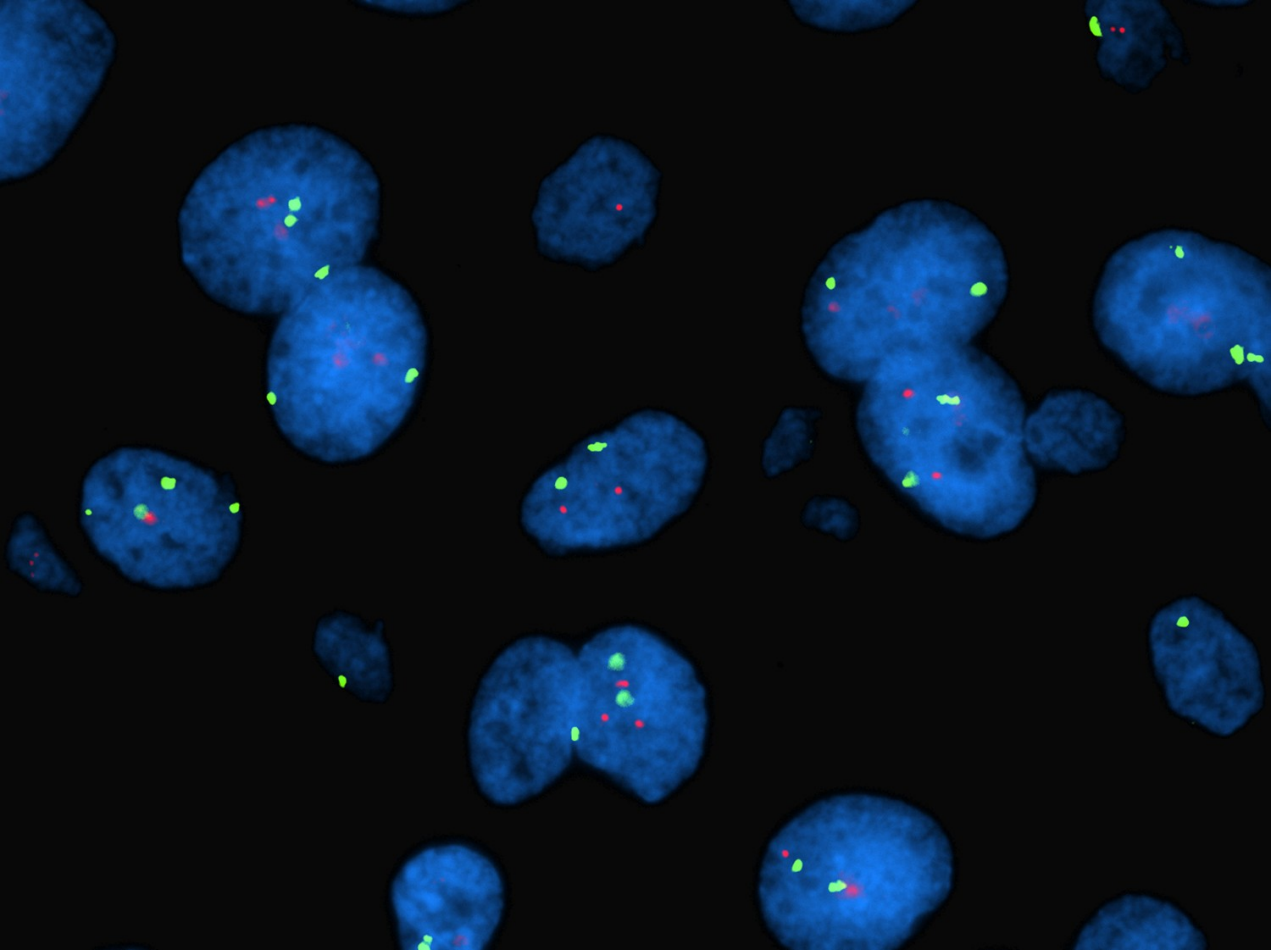
FISH, angiomatous meningioma grade I. Ratio: 1.25. The picture was taken by MBA through a Nikon eclipse 90i microscope. Green signals: centromere 17. Red signals: HER2.

*HER2* gene amplification was not detected in the FISH analysis (n = 184). Two cases were excluded due to loss of tissue. The mean and median ratios were 1.087 and 1.090 respectively, with a range of 0.76 (min: 0.82, max.: 1.58). Fig 2 shows typical FISH signals.

### Survival analysis

Only high expression of the 6B12 antibody was significantly associated with survival, as increased TTR and OS hazard ratios were observed in both uni- and multivariable survival analyses (Table 4). The expression of the other HER2 antibodies was not significantly related to any survival endpoints. Among clinical data, age was a significant prognostic factor for both endpoints. For TTR, Simpson resection grade was also significant. The assumption of proportional hazard seemed to be violated when data were stratified over Simpson grades for the OS analysis. Therefore, we carried out a supplementary analysis for OS with Simpson resection grade as a time dependent covariate (proportional with time). With this covariate, the results for the main variable (SI of 6B12) were approximately the same (HR 1.703, CI (1.231 to 2.356), p = 0.001).

**Table 4:** Cox proportional hazard regression.

**Univariable Cox proportional hazard regression**.				
**Endpoint**	**Antibody**	**HR**	**95% CI**	**p-value**
TTR (61 events)	CB11	1.030	0.888 to 1.194	0.698
TTR (61 events)	3B5	1.036	0.866 to 1.240	0.699
TTR (61 events)	SP3	1.076	0.919 to 1.255	0.368
TTR (61 events)	6B12	**1.512**	**1.132 to 2.019**	**0.005**
OS (66 events)	CB11	0.913	0.804 to 1.036	0.158
OS (67 events)	3B5	1.001	0.849 to 1.180	0.991
OS (67 events)	SP3	1.023	0.875 to 1.196	0.776
OS (66 events)	6B12	**1.397**	**1.027 to 1.900**	**0.033**
**Multivariable Cox proportional hazard regression for 6B12 (p-HER2)**.				
**Endpoint**	**Covariate**	**HR**	**95% CI**	**p-value**
TTR	SI	**1.568**	**1.153 to 2.132**	**0.004**
	Simpson grade	**2.520**	**1.506 to 4.218**	**<0.001**
	Age	**1.023**	**1.001 to 1.045**	**0.038**
	WHO performance status	1.363	0.726 to 2.560	0.335
	WHO malignancy grade	1.533	0.910 to 2.584	0.108
OS	SI	**1.679**	**1.213 to 2.323**	**0.002**
	Simpson grade	1.275	0.749 to 2.171	0.371
	Age	**1.087**	**1.061 to 1.113**	**<0.001**
	WHO performance status	1.475	0.794 to 2.741	0.219
	WHO malignancy grade	0.872	0.509 to 1.494	0.618

HR: hazard ratio (exp(B)). CI: confidence interval (95% CI for exp(B)).

### Interrater agreement

The weighted kappa statistic revealed high levels of interrater agreement for the expression of the intracellular antibodies CB11 (0.714) and 3B5 (0.579). It was much lower, however, for the phosphorylated receptor with 6B12 (0.006) and for the extracellular domain antibody SP3 (0.044).

### Raw data

All raw data are presented in S1 File.

## Discussion

The expression of HER2 was assessed in our series of human meningiomas with antibodies reactive against intracellular and extracellular domains as well as against the activated receptor. Immunoreactivity was strongest and most widely distributed for the antibodies targeting the intracellular domain, while the extracellular domain and the activated receptor were either negative or weak in expression. No relation between HER2 expression and tumor grade was observed. In the survival analyses, only high expression of the activated receptor was associated with decreased time to recurrence and overall survival. No *HER2* gene amplification was found.

All meningiomas displayed immunoreactivity for both CB11 and 3B5 antibodies targeting the intracellular domain. Other studies report frequencies ranging from 8.33% to 88.3% [11, 13, 15, 18, 19]. However, Abdelzaher et al. only investigated benign meningiomas [11], and another study only reported overexpression, defined as moderate to strong intensity of the receptor [10]. In a previous study on human astrocytomas, CB11 unveiled half as many positive cases as 3B5 [26]. Both antibodies have been regarded acceptable for use on paraffin sections [27]. We have no obvious explanation for the discrepancy of immunoreactivity between meningiomas and astrocytomas for these two antibodies.

Concerning the extracellular domain, we found mostly low expression of SP3 in about half of the tumors while the rest were negative. Published data are contradictory to our findings as they report 100% positivity and frequently with high intensity [14]. Schlegel et al. also confirmed their findings by Western blotting [14]. The reason for these discrepancies is uncertain, however, we speculate whether it could be due to shedding of the ECD [6]. Thus, there is a clear difference in expression between the extracellular and intracellular domains of HER2 in our material, and this issue needs further investigation.

Phosphorylated HER2 displayed few immunoreactive cases, and the expression level was also generally weak. We did not find any relation between expression and tumor grade. Hilton et al., however, found a decrease in staining intensity in atypical and anaplastic meningiomas compared with benign types for p-HER2 [28]. As our results, they observed mostly cytoplasmic reactivity with only a few cases exhibiting membranous staining [28]. They also found high expression of downstream signaling molecules of the HER2 receptor, “suggesting that expression is functionally significant” [28]. We found that increased expression of phosphorylated HER2 was associated with poorer survival, which could be explained by unfavorable repercussions of cellular responses to activation. However, due to its low expression level, this finding is uncertain and needs validation in larger studies. The observed discrepancies in antibody reactivity might be due to inherent aspects of immunohistochemistry (antigenicity, different antibodies, working dilutions, incubation times, etc.) and interpretation of the immunostainings.

In our study, non-neoplastic meningeal tissue adjacent to tumor tissue did not display observable immunoreactivity with any of the antibodies. However, high expression of the extracellular domain has been reported by others [14], and Chozick et al. made similar findings in their study [17]. Regarding the intracellular domain, this has previously been reported as immunonegative [13, 18] or with very low expression in some cases [15]. Further, we did not find reactivity of phosphorylated HER2 in these tissues. However, RT-PCR analyses have previously yielded high HER2 values in meningiomas [16], and higher expression than in non-neoplastic meninges [29]. Accordingly, HER2 may be expressed in normal meninges at a low level not detectable by our immunohistochemical analyses. Nevertheless, our findings are in line with the literature supporting an upregulation and activation of HER2 in human meningiomas compared with normal meninges. Whether this is a cause or result of tumor development remains to be shown. In any case, the strong immunostaining with CB11 and 3B5 in meningiomas points to a practical use of these antibodies to distinguish neoplastic from normal meningeal tissue. Further, these antibodies may have potential in targeted radiological investigation and therapeutic interventions.

None of the tested HER2 antibodies showed any association to malignancy grade, in accordance with other results [12, 14, 19]. Interestingly, Chozik et al found that expression was higher in benign tumors [17]. However, a recent study on meningioma cell lines found that HER2 overexpressing malignant meningiomas were more proliferative and invasive and had decreased apoptosis [30], suggesting a role of HER2 in aggressive tumor specimens. This is relatable to findings demonstrating that HER2 overexpression is related to worse prognosis in gastric- and breast cancers [31, 32].

In both uni- and multivariable survival analyses, only increased expression of phosphorylated receptors implied decreased TTR and OS. Others have found similar results for the intracellular domain, but only in unadjusted survival analyses [11], whereas others did not find any association of HER2 to survival [10]. Even so, due to low staining intensity and few positive cases, our findings should be interpreted judiciously and indicate a need for further analyses. However, low Simpson grade and increased age were identified as prognostic factors in the multivariable cox regression analyses, in accordance with previous studies [33–35].

We were unable to detect any *HER2* amplification in the FISH analyses, which is in accordance with previous results on southern and slot blots [14, 15, 17]. However, a few studies using FISH have found a few amplified tumors [13, 36]. Interestingly, Loussouarn et al. found that 4/10 tumors were amplified, and these tumors also showed the largest protein expression [13]. Özer et al. found only 7/55 amplified tumors, five of which were grade I [36]. Based on these findings, *HER2* gene amplification seems to be a rare event in human meningiomas.

The strength of this study is that it is population based and includes a high number of patients and long follow-up data. Among weaknesses are its retrospective nature, the inherent problems of the immunohistochemical procedure and the subjective assessments. However, diverging interpretations of the immunostainings were concluded by an experienced neuropathologist. Further, regarding inter-laboratory variations, some authors only evaluated membranous positivity [12, 13], while we made no such limitation in our material. Membranous [14, 17], cytoplasmic [18, 28], and both staining patterns for HER2 [15] have previously been reported. Heterogenous tumor tissue is a challenge using TMA, however, three extracted tissue cores from representative tumor tissue may circumvent these problems [37–39]. Further, we experienced differences in immunostaining intensity between whole sections and digitally scanned TMA tissue that also could be a source of error in the evaluation procedure. The accuracy of radiological evaluations concerning recurrence depended on technology that was probably inferior to modern diagnostics. The source of information for the degree of tumor resection largely depended on the surgeon’s descriptions from patient records, so the quality of these data may not be optimal. Additionally, some tumors were subject to therapeutic irradiation, which we have not considered in our statistical analyses. Concerning HER2 signaling activity, high protein expression and gene amplification are two possible contributing mechanisms. However, receptor mutations, constitutive activation, and ligand overexpression may also contribute, which our investigations have not addressed [5]. Summarized, our immunohistochemical investigation on HER2 expression has potential drawbacks limiting any definite conclusion on its diagnostic and prognostic value. Regardless, the variable expression levels of antibodies targeting the different domains is interesting. This aspect should be clarified and investigated for relation to prognosis in future studies.

In conclusion, we have found a generally upregulated HER2 expression in human meningiomas. Only expression of the activated/phosphorylated receptor showed any clinical relevance as it was associated with decreased time to recurrence and overall survival. This expression pattern may be influential on prognostication and therapeutic decision making of meningioma patients and needs to be further elucidated.

## Supporting information

S1 Fig HER2 controls. The picture was taken by SHT with a Nikon eclipse 80i microscope, Lumenera’s Infinity2 camera, and Infinity Analyze software. Magnification: 400x. Microsoft Paint was used to create the final composite image. a. Negative control, Paget’s disease b. Positive control for 6B12, Paget’s disease.
(TIF)

S1 Table Differences in SI according to tumor localization and subtype (overall p-values, Kruskal-Wallis tests). (DOCX)

S1 File Raw data. (XLSX)
