# Supplementary figures and images for: Prognostic value of ErbB2/HER2 in human meningiomas
Source: PLoS One. 2018 Oct 18;13(10):e0205846. doi: 10.1371/journal.pone.0205846 (PMC6193666; doi:10.1371/journal.pone.0205846)

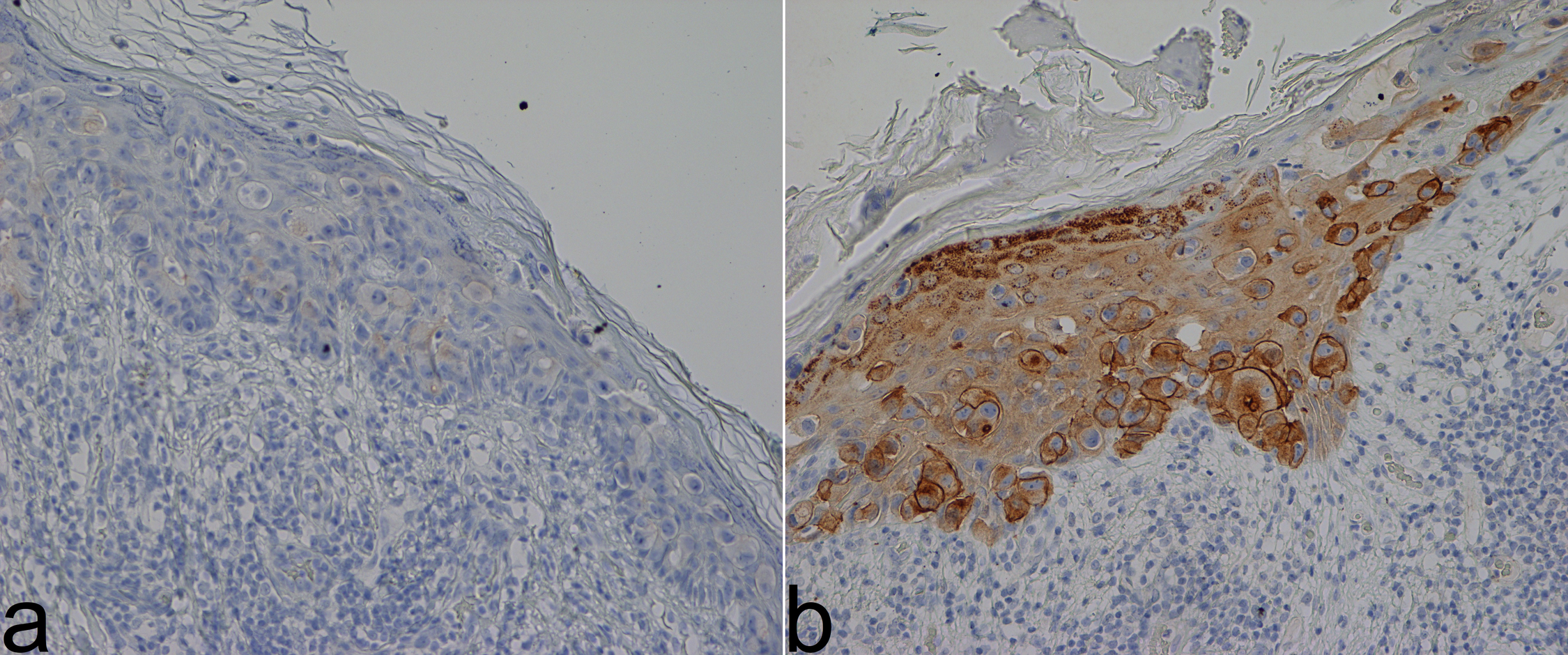

Supplement: S1 Fig — The picture was taken by SHT with a Nikon eclipse 80i microscope, Lumenera’s Infinity2 camera, and Infinity Analyze software. Magnification: 400x. Microsoft Paint was used to create the final composite image. a. Negative control, Paget’s disease b. Positive control for 6B12, Paget’s disease. (TIF) [file pone.0205846.s001.tif]
