# Supplementary material for: Prognostic value of ErbB2/HER2 in human meningiomas
Source: PLoS One. 2018 Oct 18;13(10):e0205846. doi: 10.1371/journal.pone.0205846 (PMC6193666; doi:10.1371/journal.pone.0205846)
Supplement: S1 Table — (DOCX) [file pone.0205846.s002.docx]

|  | CB11 | 3B5 | SP3 | Tyr1221/1222 |
| --- | --- | --- | --- | --- |
| Tumor localization | 0.092 | 0.197 | 0.080 | 0.390 |
| Tumor subtype | 0.802 | 0.178 | 0.520 | 0.103 |

**S1 Table. Differences in SI according to tumor localization and subtype (overall p-values, Kruskal-Wallis tests).**
